# Anxiety and depression symptoms after COVID-19 infection: results from the COVID Symptom Study app

**DOI:** 10.1101/2021.07.07.21260137

**Published:** 2021-07-08

**Authors:** Kerstin Klaser, Ellen J. Thompson, Long H. Nguyen, Carole H. Sudre, Michela Antonelli, Benjamin Murray, Liane S. Canas, Erika Molteni, Mark S. Graham, Eric Kerfoot, Liyuan Chen, Jie Deng, Anna May, Christina Hu, Andy Guest, Somesh Selvachandran, David A Drew, Marc Modat, Andrew T. Chan, Jonathan Wolf, Tim D. Spector, Alexander Hammers, Emma L. Duncan, Sebastien Ourselin, Claire J. Steves

**Affiliations:** 1 School of Biomedical Engineering & Imaging Sciences, King’s College London, London SE1 7EU, UK; 2 Department of Twin Research and Genetic Epidemiology, King’s College London, London SE1 7EH, UK; 3 Clinical & Translational Epidemiology Unit, Massachusetts General Hospital and Harvard Medical School, 100 Cambridge Street, Boston, MA, USA; 4 Division of Gastroenterology, Department of Medicine, Massachusetts General Hospital and Harvard Medical School, 100 Cambridge Street, Boston, MA, USA; 5 MRC Unit for Lifelong Health and Ageing, Department of Population Science and Experimental Medicine, University College London, UK; 6 Centre for Medical Image Computing, Department of Computer Science, University College London, UK; 7 King’s College London & Guy’s and St Thomas’ PET Centre, London, UK; 8 Zoe Limited, London SE1 7RW, UK

**Keywords:** COVID-19, SARS-CoV-2, Mental health, Anxiety, Depression, GAD2, PHQ2

## Abstract

**Background:**

Mental health issues have been reported after SARS-CoV-2 infection. However, comparison to prevalence in uninfected individuals and contribution from common risk factors (e.g., obesity, comorbidities) have not been examined. We identified how COVID-19 relates to mental health in the large community-based COVID Symptom Study.

**Methods:**

We assessed anxiety and depression symptoms using two validated questionnaires in 413,148 individuals between February and April 2021; 26,998 had tested positive for SARS-CoV-2. We adjusted for physical and mental pre-pandemic comorbidities, BMI, age, and sex.

**Findings:**

Overall, 26.4% of participants met screening criteria for general anxiety and depression. Anxiety and depression were slightly more prevalent in previously SARS-CoV-2 positive (30.4%) vs. negative (26.1%) individuals. This association was small compared to the effect of an unhealthy BMI and the presence of other comorbidities, and not evident in younger participants (≤40 years). Findings were robust to multiple sensitivity analyses. Association between SARS-CoV-2 infection and anxiety and depression was stronger in individuals with recent (<30 days) vs. more distant (>120 days) infection, suggesting a short-term effect.

**Interpretation:**

A small association was identified between SARS-CoV-2 infection and anxiety and depression symptoms. The proportion meeting criteria for self-reported anxiety and depression disorders is only slightly higher than pre-pandemic.

## Introduction

Studies from previous coronaviruses suggesting an increased risk of neurological disorders^1^, and case studies^2-4^ and findings^5-7^ regarding the impact of SARS-CoV-2 infection on the central nervous system^8-11^ led to the hypothesis that anxiety/depression symptoms may be more prevalent in individuals after SARS-CoV-2 infection. Indeed, several reports suggest that COVID-19 survivors are at increased risk of mood and anxiety disorders three months post-infection^12-15^. Moreover, the Office for National Statistics reported a steep increase in anxiety/depression symptoms in the general public (irrespective of infection status) compared to pre-pandemic data, adjusting for socioeconomic factors^16^.

Quantifying the relationship of SARS-CoV-2 infection on anxiety/depression symptoms *per se* requires disentangling the consequences of infection from other factors such as lockdown measures. Direct links to health records may enable assessment of SARS-CoV-2 infection on psychiatric diagnoses^15^; however, it takes time and resources to acquire large cohorts for such longitudinal studies. Alternatively, analysis of self-reported real-time data allows for faster and timely insights into effects on mental health from SARS-CoV-2 infections.

This study aimed to assess prevalence of anxiety/depression symptoms in individuals with and without prior SARS-CoV-2 infection using a large community cohort, including assessment of other known mental health predictors. We used data from 413,148 tested non-healthcare workers who answered a mental health survey between February and April 2021 via the COVID Symptom Study app^17^.

## Methods

### Sample

Data were acquired by the COVID Symptom Study app^17^, a mobile application developed by health data company Zoe Limited in collaboration with King’s College London (KCL), the Massachusetts General Hospital, Lund University, and Uppsala University. The app was launched on 24 March 2020 and allows users to report their health status (whether symptomatic or asymptomatic), SARS-CoV-2-related testing and results, and vaccination details, daily. Upon registration, app users provide demographic and clinical data including age, height, weight, sex, comorbidities (i.e., cancer, diabetes, eczema, heart disease, lung disease, kidney disease and hay fever), and healthcare worker status. The app contents can also be modified to address arising research questions. We used data from 413,148 non-healthcare worker users who answered a mental health survey between February and April 2021, and reported a SARS-CoV-2 test result. Healthcare workers were excluded from this analysis due to their likely differing pandemic experience.

### Measures

Between 23 February 2021 and 12 April 2021 app contributors were invited to answer a survey about their mental health. Anxiety/depression symptoms were measured using the Generalised Anxiety Disorder assessment-2 (GAD-2)^18^ and the Patient Health Questionnaire-2 (PHQ-2)^19^. These measures examine symptoms in the preceding two weeks, each using two questions. For each question regarding frequency of a proposed situation/feeling, users can answer “Not at all”, “Several days”, “More than half the days” or “Nearly every day”. Each answer scores from 0 for “Not at all” to 3 for “Nearly every day”. Each questionnaire has a score ranging from 0-6. Previous studies have shown an optimal cut-off point for possible anxiety or depression disorder of ≥3, yielding a sensitivity of >80%^18,19^. A binary outcome variable was created by grouping those who scored ≥3 in either GAD-2 or PHQ-2, or <3.

Logistic regression was used to study whether mental health status was associated with a positive SARS-CoV-2 test result. We adjusted for age, sex, body mass index (BMI) groups (underweight, overweight, obese, with normal BMI (18.5-24.9) as the reference), and comorbidities including learning disabilities; and applied inverse probability weighting for the probability of getting tested for SARS-CoV-2. Data are presented using descriptive statistics. Additionally, logistic regressions were employed stratifying by age groups (18-30, 31-40, 41-50, 51-60, 61-70, >70). A sensitivity analysis was performed stratifying by presence of pre-pandemic mental health disorders (see Supplementary Materials).

Further analyses in SARS-CoV-2 affected individuals assessed for association with days since infection confirmation and anxiety/depression symptoms, using days between positive test date and date of survey, grouped into: <30, 30-60, 60-90, 90-120, and >120 days. Individuals reporting a positive test result >120 days before answering the survey served as reference.

Data were extracted and pre-processed with ExeTera^20^, a Pandas-like library developed at KCL, and statistical analysis was performed using Python (Pandas, NumPy and SciPy).

Ethical approval for use of the app for research purposes in the UK was granted by the KCL Ethics Committee (review reference LRS-19/20-18210); all users provided consent for non-commercial use.

## Results

Between 23 February and 12 April 2021, 421,977 non-healthcare workers (aged 18-99 years, BMI 15-55) answered the mental health survey and logged a SARS-CoV-2 test result (386,150 negative, 35,827 positive). 26,998 positive tests were PCR or lateral flow results; positive antibody tests (8,829) were excluded as time of infection was unknown.

26.4% (109,116) of participants scored ≥3 in GAD2 and/or PHQ2. Participants with anxiety/depression symptoms were younger, had more comorbidities, and were more often female, compared to unaffected individuals. Among those predicted to have anxiety or depression (based on a score of ≥3 on GAD-2 or PHQ-2), 38.06% (41,525) reported a previous diagnosis of a pre-pandemic mental health disorder and 5.79% (6,320) a learning disability. The study population’s demographic characteristics are presented in Table 1.

**Figure 1:**
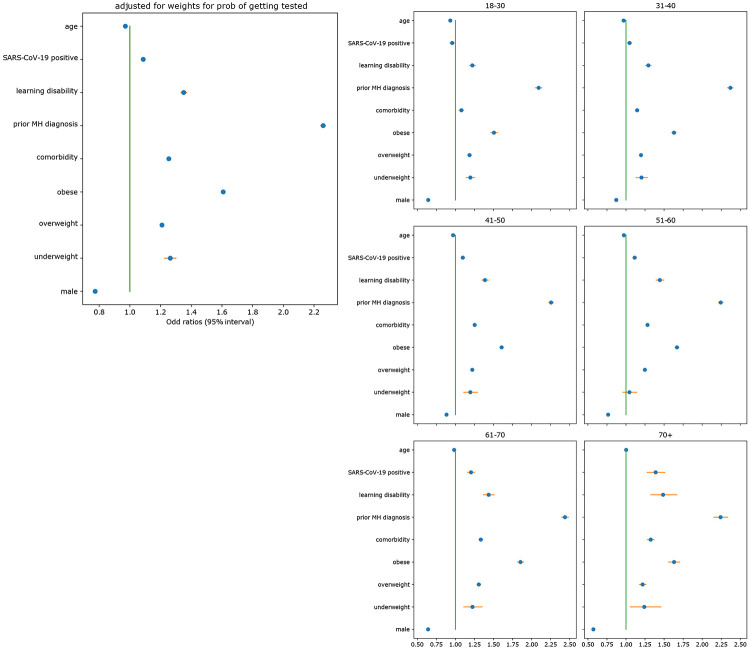
Association between age, BMI, male sex, comorbidities, a previous diagnosis of a mental health condition, learning disabilities, a positive SARS-CoV-2 test result (PCR and lateral flow), and the odds ratio of anxiety/depression symptoms suggested by the results of the mental health survey.

SARS-CoV-2 infection was associated with anxiety/depression symptoms (OR 1.08, 95% CI [1.07 –1.10], p<0.001). However, stronger associations with anxiety/depression symptoms were observed for unhealthy BMI categories (i.e., underweight, overweight, obese) with odds ratios of 1.26 ([1.22 –1.30], p<0.001), 1.21 ([1.20 –1.22], p<0.001), and 1.61 ([1.59 –1.62], p<0.001), respectively. Participants reporting one or more comorbidities (OR 1.25, 95% CI [1.24,1.26], p<0.001), and those with learning disabilities (OR 1.35, 95% CI [1.33 –1.37], p<0.001) were more likely to have anxiety/depression symptoms. Individuals reporting a previously diagnosed mental health condition had the highest odds of reporting anxiety/depression symptoms (OR 2.26, 95% CI [2.24 – 2.28], p<0.001) (Figure 1).

We observed no significant difference in the small overall increased odds of anxiety/depression symptoms with SARS-CoV-2 infection in those with a history of prior mental health conditions (OR 1.09, 95% CI [1.06 – 1.12], p <0.001); and those without such prior history of 1.09, 95% CI [1.07 – 1.10], p<0.001).

Stratification by age group showed no association between a positive SARS-CoV-2 test and anxiety/depression symptoms in young groups (<40 years). Other variables (sex, comorbidities, BMI) were consistent in age-stratified analyses. Finally, in the 26,998 cases positive for SARS-CoV-2 by PCR and lateral flow, we tested whether elapsed time after a positive test affected mental health. The relationship between SARS-CoV-2 and depression/anxiety symptoms changed over time, with increased risk of anxiety/depression symptoms in those diagnosed <30 days compared to those diagnosed >120 days prior to the survey (OR 1.15, 95% CI [1.10 - 1.2], p<0.001).

## Discussion

In this large, community-based study, we report a small positive association between SARS-CoV-2 infection and anxiety/depression symptoms. However, this was dwarfed by associations with the known risk factors BMI, sex, and comorbidities. Results were robust to sensitivity analyses stratifying by prior mental health disorder diagnoses. Further, no association between SARS-CoV-2 infection and anxiety/depression symptoms was found in younger age groups (<40 years).

Association between SARS-CoV-2 infection and anxiety/depression symptoms changed over time, with the strongest association in those infected <30 days prior to the survey, suggesting a short-term effect of infection on mental health only. It is possible that other factors affecting mental health which were changing over the pandemic (e.g., lockdown) may moderate an effect of elapsed time since SARS-CoV-2 infection on mental health^21^.

Overall prevalence of anxiety/depression symptoms in our study (26.4%) is slightly increased compared to pre-pandemic levels of mental health issues in the UK general population assessed by the UK Household Longitudinal Study with the GHQ-12 questionnaire (18.9% in 2018^22^) but broadly comparable to the level seen in April 2021 (27.3%^22^). This previous 2021 study did not explore any relationship with SARS-CoV-2 infection. A recent analysis of 1112 subjects experiencing probable COVID-19 symptoms suggested a positive association between COVID-19 and anxiety/depression symptoms 1-7 months after suggested infection (OR 1.31 - 1.47)^23^. Our study benefits from a much larger sample size of tested participants.

Our study has several limitations. Data are self-reported using a mobile app, and may disproportionately represent more affluent populations. We only had one time point of mental health data collection, limiting our ability to test if associations changed as the pandemic progressed. Additionally, although we applied weighting for the probability of being tested for the virus, results referring to time since testing might be still biased due to limited testing capacity early in the pandemic. As in any study assessing mental health through questionnaires, selection bias (whereby mental health influences who responds) and reporting bias (relating to perception, and/or influence of a ‘valid’ reason to report) may limit the validity of our results. Further analyses of longitudinal datasets with different reporting structures are warranted.

## Conclusion

This study suggests a weak association between SARS-CoV-2 infection and anxiety/depression symptoms, especially in adults >40 years, which is small relative to known risk factors such as previous medical or mental health conditions and/or unhealthy BMI. The association was most evident in recently infected individuals. This suggests that an effect of SARS-CoV-2 on mental health may be only of short duration. Further exploration may help to understand factors that will improve mental health after SARS-CoV-2 infection.

## Supplementary Material

Supplement 1

## Figures and Tables

**Table 1:** Demographic characteristics table

	Anxiety or Depression symptoms	Anxiety or Depression symptoms	Total
	YES	NO	n = 413148
	n = 109116 (26.4%)	n = 304032 (73.6%)	
**Age, years (mean, standard deviation)**	50.7 (13.9)	55.6 (12.9)	54.1 (13.4)
**Underweight (BMI < 18.5)**	1459 (1.34%)	3190 (1.05%)	4649 (1.13%)
**Normal weight (BMI 18.6 - 24.9)**	40816 (37.41%)	134695 (44.30%)	175511 (42.48%)
**Overweight (BMI 25 - 29.9)**	36012 (33.00%)	106455 (35.01%)	142467 (34.48%)
**Obese (BMI > 30)**	30829 (28.25%)	59692 (19.63%)	90521 (21.91%)
**Has Comorbidity**	22006 (20.17%)	52558 (17.29%)	74564 (18.05%)
**Has No Comorbidity**	87110 (79.83%)	251474 (82.71%)	338584 (81.95%)
**Previously diagnosed with mental health condition**	41525 (38.06%)	57067 (18.77%)	98592 (23.86%)
**No previous mental health condition**	67591 (61.94%)	246965 (81.23%)	314556 (76.14%)
**Learning disability YES**	6320 (5.79%)	9987 (3.28%)	16307 (3.95%)
**Learning disability NO**	102796 (94.21%)	294045 (96.72%)	396841 (96.05%)
**Female**	80446 (73.73%)	200563 (65.97%)	281009 (68.02%)
**Male**	28670 (26.27%)	103469 (34.03%)	132139 (31.98%)
**Negative COVID-19 Test**	100897 (92.47%)	285253 (93.82%)	386150 (93.47%)
**Positive COVID-19 Test**	8219 (7.53%)	18779 (6.18%)	26998 (6.53%)
**Positive COVID-19**	1314 (15.99%)	2480 (13.21%)	3794 (14.05%)
**<30 days before survey**			
**Positive COVID-19**	2149 (26.15%)	5043 (26.85%)	7192 (26.64%)
**30-60 days before survey**			
**Positive COVID-19**	1843 (22.42%)	4505 (23.99%)	6348 (23.51%)
**60-90 days before survey**			
**Positive COVID-19**	1162 (14.14%)	2791 (14.86%)	3953 (14.64%)
**90-120 before survey**			
**Positive COVID-19**	1751 (21.30%)	3960 (21.09%)	5711 (21.15%)
**<120 before survey**			

## References

[1] Rogers Jonathan P., "Psychiatric and neuropsychiatric presentations associated with severe coronavirus infections: a systematic review and meta-analysis with comparison to the COVID-19 pandemic." The Lancet Psychiatry 7.7 (2020): 611–627. 32437679 10.1016/S2215-0366(20)30203-0 PMC7234781

[2] Ellul Mark A., "Neurological associations of COVID-19." The Lancet Neurology (2020). 10.1016/S1474-4422(20)30221-0 PMC7332267 32622375

[3] Varatharaj Aravinthan, "Neurological and neuropsychiatric complications of COVID-19 in 153 patients: a UK-wide surveillance study." The Lancet Psychiatry 7.10 (2020): 875–882. 32593341 10.1016/S2215-0366(20)30287-X PMC7316461

[4] Paterson Ross W., "The emerging spectrum of COVID-19 neurology: clinical, radiological and laboratory findings." Brain 143.10 (2020): 3104–3120. 32637987 10.1093/brain/awaa240 PMC7454352

[5] Kremer Stéphane, "Neurologic and neuroimaging findings in patients with COVID-19: A retrospective multicenter study." Neurology 95.13 (2020): e1868–e1882. 32680942 10.1212/WNL.0000000000010112

[6] Pezzini Alessandro, and Padovani Alessandro. "Lifting the mask on neurological manifestations of COVID-19." Nature Reviews Neurology 16.11 (2020): 636–644. 32839585 10.1038/s41582-020-0398-3 PMC7444680

[7] Raman Betty, "Medium-term effects of SARS-CoV-2 infection on multiple vital organs, exercise capacity, cognition, quality of life and mental health, post-hospital discharge." EClinicalMedicine 31 (2021): 100683. 33490928 10.1016/j.eclinm.2020.100683 PMC7808914

[8] Iadecola Costantino, Anrather Josef, and Kamel Hooman. "Effects of COVID-19 on the nervous system." Cell (2020). 10.1016/j.cell.2020.08.028 PMC7437501 32882182

[9] Kreye Jakob, Momsen Reincke S., and Prüss Harald. "Do cross-reactive antibodies cause neuropathology in COVID-19?." Nature Reviews Immunology 20.11 (2020): 645–646. 10.1038/s41577-020-00458-y PMC7537977 33024283

[10] Meinhardt Jenny, "Olfactory transmucosal SARS-CoV-2 invasion as a port of central nervous system entry in individuals with COVID-19." Nature neuroscience 24.2 (2021): 168–175. 33257876 10.1038/s41593-020-00758-5

[11] Rhea Elizabeth M., "The S1 protein of SARS-CoV-2 crosses the blood-brain barrier in mice." Nature neuroscience 24.3 (2021): 368–378. 33328624 10.1038/s41593-020-00771-8 PMC8793077

[12] Holmes Emily A., "Multidisciplinary research priorities for the COVID-19 pandemic: a call for action for mental health science." The Lancet Psychiatry (2020). 10.1016/S2215-0366(20)30168-1 PMC7159850 32304649

[13] Vindegaard Nina, and Benros Michael Eriksen. "COVID-19 pandemic and mental health consequences: Systematic review of the current evidence." Brain, behavior, and immunity 89 (2020): 531–542. 10.1016/j.bbi.2020.05.048 PMC7260522 32485289

[14] Taquet Maxime, "6-month neurological and psychiatric outcomes in 236 379 survivors of COVID-19: a retrospective cohort study using electronic health records." The Lancet Psychiatry (2021). 10.1016/S2215-0366(21)00084-5 PMC8023694 33836148

[15] Taquet Maxime, "Bidirectional associations between COVID-19 and psychiatric disorder: retrospective cohort studies of 62 354 COVID-19 cases in the USA." The Lancet Psychiatry 8.2 (2021): 130–140. 33181098 10.1016/S2215-0366(20)30462-4 PMC7820108

[16] Office for National Statistics, “Coronavirus and depression in adults, Great Britain: January to March 2021”, https://www.ons.gov.uk/peoplepopulationandcommunity/wellbeing/articles/coronavirusanddepressioninadultsgreatbritain/januarytomarch2021

[17] Menni Cristina, "Real-time tracking of self-reported symptoms to predict potential COVID-19." Nature medicine 26.7 (2020): 1037–1040. 10.1038/s41591-020-0916-2 PMC7751267 32393804

[18] Kroenke Kurt, "Anxiety disorders in primary care: prevalence, impairment, comorbidity, and detection." Annals of internal medicine 146.5 (2007): 317–325. 17339617 10.7326/0003-4819-146-5-200703060-00004

[19] Kroenke Kurt, Spitzer Robert L., and Williams Janet BW. "The Patient Health Questionnaire-2: validity of a two-item depression screener." Medical care (2003): 1284–1292. 14583691 10.1097/01.MLR.0000093487.78664.3C

[20] Murray Benjamin, "Accessible data curation and analytics for international-scale citizen science datasets." arXiv preprint arXiv:2011.00867 (2020). 10.1038/s41597-021-01071-x PMC8608807 34811392

[21] O'Connor R., Wetherall K., Cleare S., McClelland H., Melson A., Niedzwiedz C., … Robb K. (2021). Mental health and well-being during the COVID-19 pandemic: Longitudinal analyses of adults in the UK COVID-19 Mental Health & Wellbeing study. The British Journal of Psychiatry, 218(6), 326–333. 10.1192/bjp.2020.212 PMC7684009 33081860

[22] Pierce Matthias, "Mental health before and during the COVID-19 pandemic: a longitudinal probability sample survey of the UK population." The Lancet Psychiatry 7.10 (2020): 883–892 32707037 10.1016/S2215-0366(20)30308-4 PMC7373389

[23] Niedzwiedz, “Psychological distress among people with probable COVID-19 infection: Analysis of the UK Household Longitudinal Study.” BJPsych Open, 7(3), (2021), E104. 34001295 10.1192/bjo.2021.63 PMC8134894

