# Supplementary material for: Anxiety and depression symptoms after COVID-19 infection: results from the COVID Symptom Study app
Source: medRxiv. 2021 Jul 8:2021.07.07.21260137. Preprint. [Version 1] doi: 10.1101/2021.07.07.21260137 (PMC8282115; doi:10.1101/2021.07.07.21260137)
Supplement: Supplement 1 [file media-1.pdf]

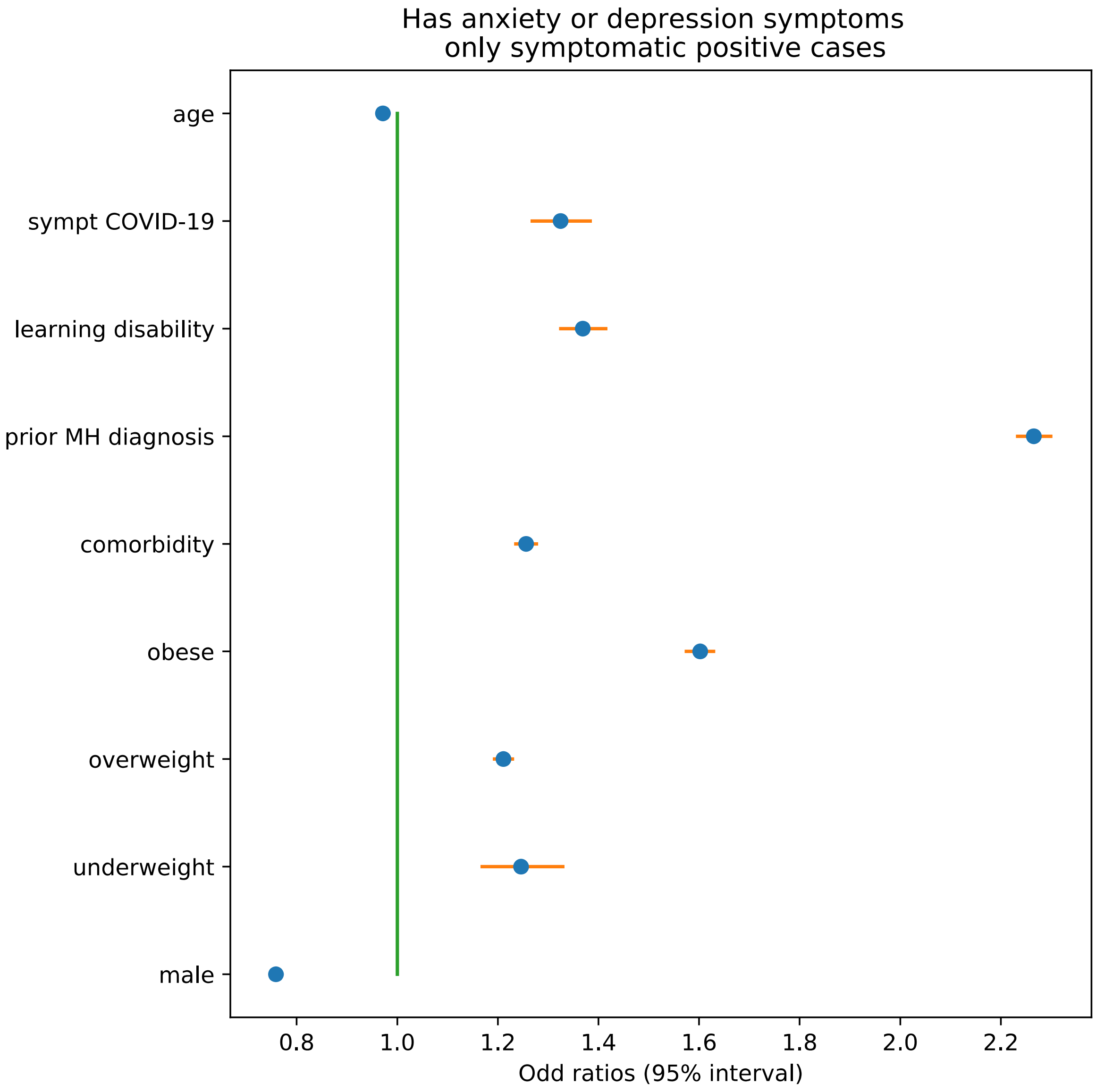

Supplementary Figure 1: Association between age, BMI, male sex, comorbidities, a previous diagnosis of a mental health (MH) condition, learning disabilities, a positive SARS-CoV-2 test result (PCR and lateral flow), and the odds ratio of anxiety/depression symptoms suggested by the results of the mental health survey. Only symptomatic cases were considered in this sensitivity analysis.

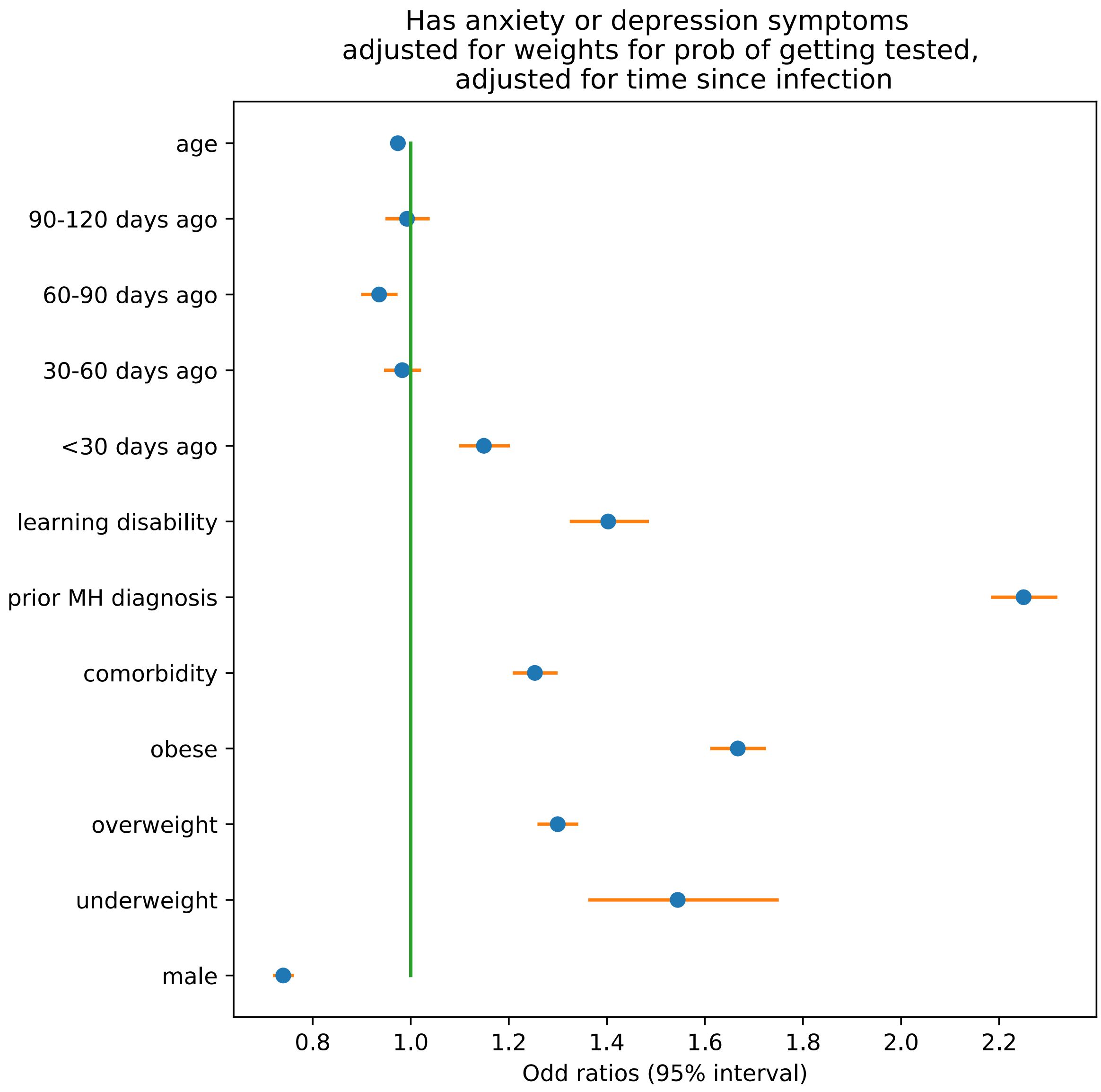

Supplementary Figure 2: Association between age, BMI, male sex, comorbidities, a previous diagnosis of a mental health (MH) condition, learning disabilities, time since infection occured, and the odds ratio of anxiety/depression symptoms suggested by the results of the mental health survey. This sensitivity analysis was performed on users with a positive SARS-CoV-2 test result (PCR and lateral flow) only.

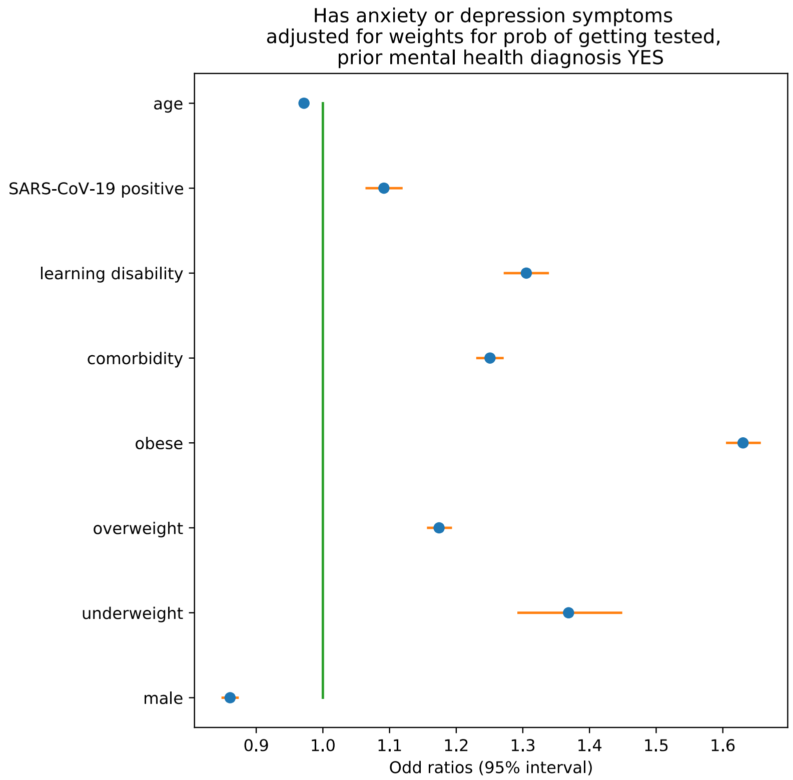

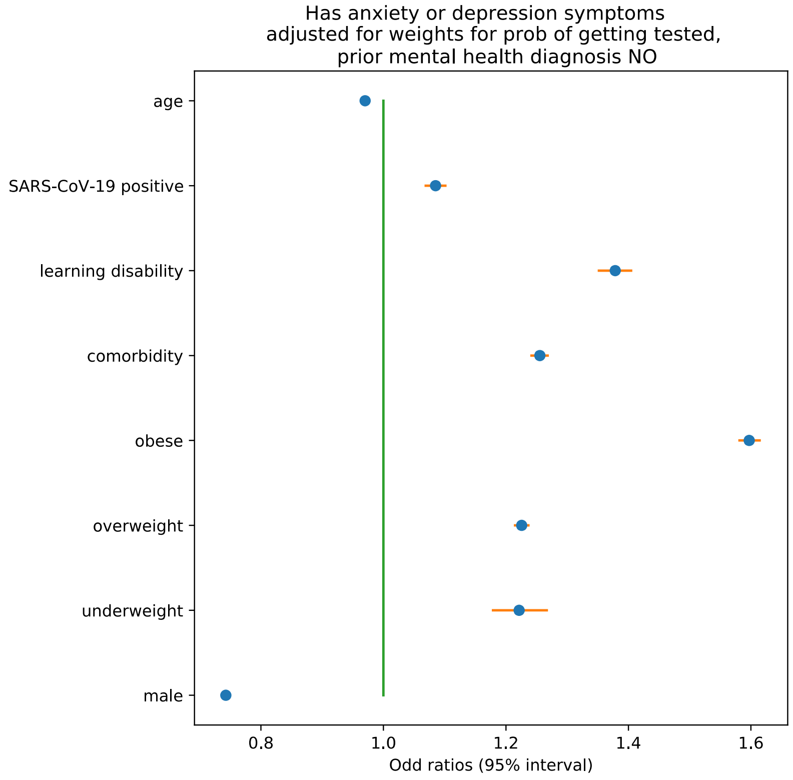

Supplementary Figure 3: Association between age, BMI, male sex, comorbidities, learning disabilities, a positive SARS-CoV-2 test result (PCR and lateral flow), and the odds ratio of anxiety/depression symptoms suggested by the results of the mental health survey stratified by prior mental health condition.

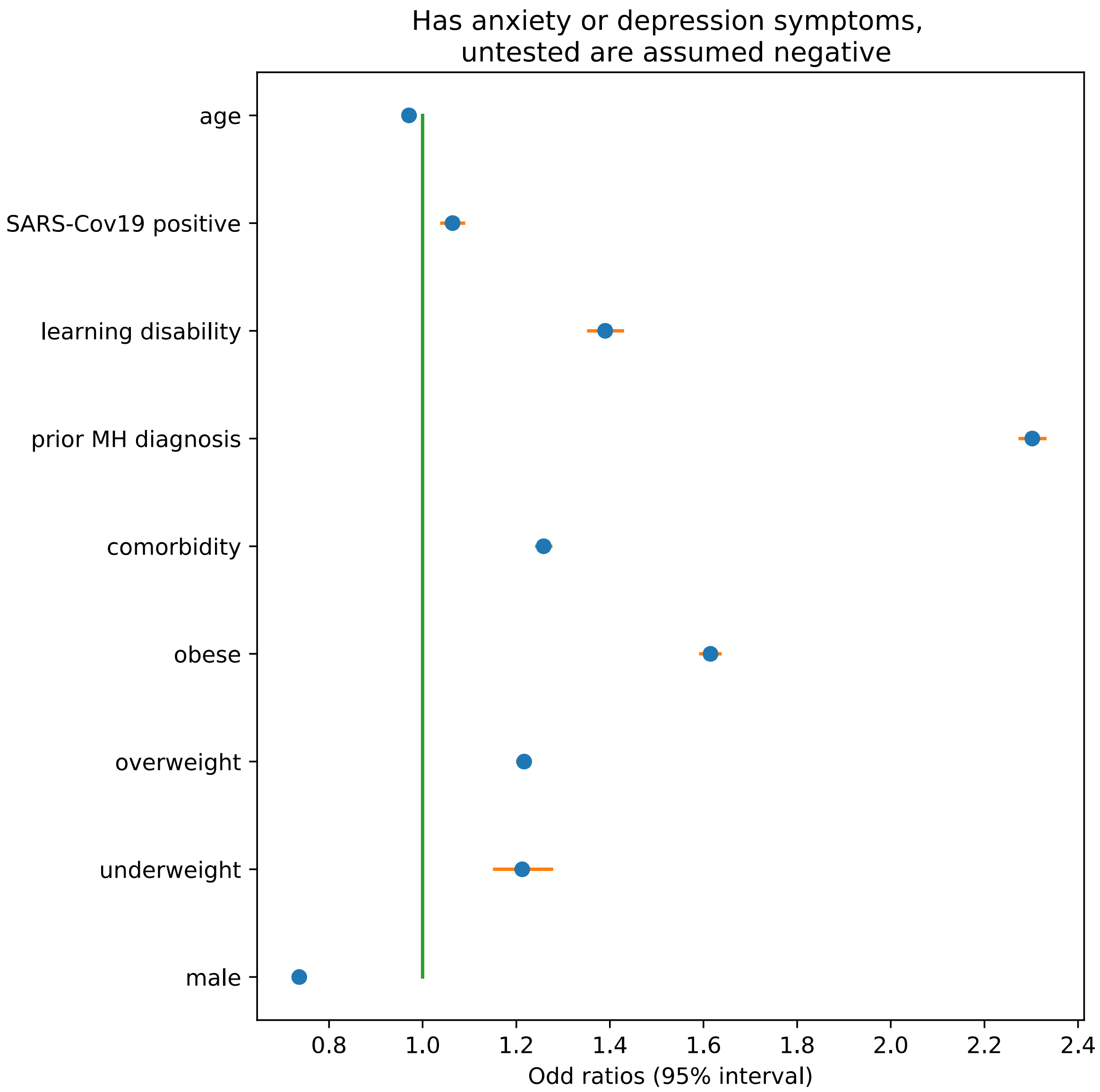

Supplementary Figure 4: Association between age, BMI, male sex, comorbidities, a previous diagnosis of a mental health (MH) condition, learning disabilities, a positive SARS-CoV-2 test result (PCR and lateral flow), and the odds ratio of anxiety/depression symptoms suggested by the results of the mental health survey. Untested cases were assumed negative test results in this sensitivity analysis.
